# 超临界流体-蒸发光散射法测定常见油脂类药用辅料皂化后的脂肪酸组成及其在油脂鉴别中的应用

**DOI:** 10.3724/SP.J.1123.2024.01003

**Published:** 2024-06-08

**Authors:** Ziying WANG, Haiwei SHI, Congyu MA, Wenyuan LIU, Lei CHEN, Zhen LIU, Yaozuo YUAN, Mei ZHANG, Sheng TANG

**Affiliations:** 1.中国药科大学药学院, 江苏 南京 211112; 1. College of Pharmacy, China Pharmaceutical University, Nanjing 211112, China; 2.江苏省食品药品监督检验研究院, 江苏 南京 210019; 2. Jiangsu Institute for Food and Drug Control, Nanjing 210019, China; 3.国家药品监督管理局化学药品杂质谱研究重点实验室, 江苏 南京 210019; 3. NMPA Key Laboratory for Impurity Profile of Chemical Drugs, Nanjing 210019, China; 4.中国医学科学院药物研究所, 北京 100050; 4. Institute of Materia Medica, Chinese Academy of Medical Sciences, Beijing 100050, China; 5.国家药典委员会, 北京 100061; 5. Chinese Pharmacopoeia Commission, Beijing 100061, China; 6.南京市食品药品监督检验院, 江苏 南京 211198; 6. Nanjing Institute for Food and Drug Control, Nanjing 211198, China; 7.江苏科技大学环境与化学工程学院, 江苏 镇江 212003; 7. College of Environmental and Chemical Engineering, Jiangsu University of Science and Technology, Zhenjiang 212003, China

**Keywords:** 超临界流体色谱, 蒸发光散射检测器, 油脂类药用辅料, 脂肪酸, 皂化反应, supercritical fluid chromatography (SFC), evaporative light-scattering detector (ELSD), oil pharmaceutical excipients, fatty acids, saponification reaction

## Abstract

脂肪酸是油脂类药用辅料的重要组成成分,可直接反映油脂类药用辅料及制剂的质量和安全性。本研究利用超临界流体色谱-蒸发光散射检测器,建立了同时测定肉豆蔻酸、棕榈酸、硬脂酸、花生酸、二十二烷酸和木蜡酸6种脂肪酸的分析方法。为避免脂肪酸与酯相互转化而造成的脂肪酸种类与含量测定偏差,本方法采取先皂化后测定的方式,并系统优化了实验条件,实现了6种脂肪酸的定量分析。在优化的实验条件下,6种脂肪酸可在12 min内实现高效分离,并且在各自的质量浓度范围内线性关系良好,检出限和定量限分别为5~10 mg/L和10~25 mg/L,加标回收率为80.93%~111.66%。本文所建立方法灵敏,高效,重复性和稳定性好,满足油脂类药用辅料中脂肪酸的分析要求,并成功应用于5种常见油脂类药用辅料(玉米油、大豆油、椰子油、橄榄油、花生油)中6种脂肪酸的含量测定;同时结合主成分分析方法,实现了对不同油脂类药用辅料的准确判别,为油脂类药用辅料的快速鉴别和掺伪分析提供了有价值的参考。

## Abstract (en)

Oils and fats are commonly used in the pharmaceutical industry as solvents, emulsifiers, wetting agents, and dispersants, and are an important category of pharmaceutical excipients. Fatty acids with unique compositions are important components of oil pharmaceutical excipients. The Chinese Pharmacopoeia provides clear descriptions of the fatty acid types and limits suitable for individual oil pharmaceutical excipient. An unqualified fatty acid composition or content may indicate adulteration or deterioration. The fatty acid composition, as a key indicator for the identification and adulteration evaluation of oil pharmaceutical excipients, can directly affect the quality and safety of oil pharmaceutical excipients and preparations. Gas chromatography is the most widely used technique for fatty acid analysis, but it generally requires derivatization, which affects quantitative accuracy. Supercritical fluid chromatography (SFC), an environmentally friendly technique with excellent separation capability, offers an efficient method for detecting fatty acids without derivatization. Unlike other chromatographic methods, SFC does not use nonvolatile solvents (e. g., water) as the mobile phase, rendering it compatible with an evaporative light-scattering detector (ELSD) for enhanced detection sensitivity. However, the fatty acids in oil pharmaceutical excipients exist in the free and bound forms, and the low content of free fatty acids in these oil pharmaceutical excipients not only poses challenges for their detection but also complicates the determination of characteristic fatty acid compositions and contents. Moreover, the compositions and ratios of fatty acids are influenced by environmental factors, leading to interconversion between their two forms. In this context, saponification provides a simpler and faster alternative to derivatization. Saponification degrades oils and fats by utilizing the reaction between esters and an alkaline solution, ultimately releasing the corresponding fatty acids. Because this method is more cost effective than derivatization, it is a suitable pretreatment method for the detection of fatty acids in oil pharmaceutical excipients using the SFC-ELSD approach. In this study, we employed SFC-ELSD to simultaneously determine six fatty acids, namely, myristic acid, palmitic acid, stearic acid, arachidic acid, docosanoic acid, and lignoceric acid, in oil pharmaceutical excipients. Saponification of the oil pharmaceutical excipients using sodium hydroxide methanol solution effectively avoided the bias in the determination of fatty acid species and contents caused by the interconversion of fatty acids and esters. The separation of the six fatty acids was achieved within 12 min, with good linearity within their respective mass concentration ranges. The limits of detection and quantification were 5-10 mg/L and 10-25 mg/L, respectively, and the spiked recoveries were 80.93%-111.66%. The method proved to be sensitive, reproducible, and stable, adequately meeting requirements for the analysis of fatty acids in oil pharmaceutical excipients. Finally, the analytical method was successfully applied to the determination of six fatty acids in five types of oil pharmaceutical excipients, namely, corn oil, soybean oil, coconut oil, olive oil, and peanut oil. It can be combined with principal component analysis to accurately differentiate different types of oil pharmaceutical excipients, providing technical support for the rapid identification and quality control of oil pharmaceutical excipients. Thus, the proposed method may potentially be applied to the analysis of complex systems adulterated with oil pharmaceutical excipients.

油脂不仅是重要的人体营养物质,还可以作为溶剂、乳化剂、润湿剂、分散剂等广泛应用于制药和药用化妆品领域^[1,2]^,属于药用辅料的重要类别^[3]^。油脂类药用辅料由甘油和不同链长及不饱和度的脂肪酸(如肉豆蔻酸、棕榈酸、硬脂酸等)构成,每种油脂类药用辅料都具有特征性的脂肪酸组成,其中脂肪酸的种类和含量可用于油脂种类的鉴别,并直接决定了油脂类药用辅料的性质、用途和产品质量^[4⇓-6]^。2020版《中国药典》^[7]^规定,硬脂酸在大豆油、玉米油和花生油中的含量应分别为2.5%~5.0%、1.0%~3.3%和1.3%~6.5%;若脂肪酸的组成或含量不合格,说明油脂存在掺假或变质等问题,例如亚麻酸等多不饱和脂肪酸的含量过高时,会导致油脂更易发生氧化变质。油脂类药用辅料作为纳米乳剂时,除了受脂肪酸不饱和度的影响,其药物功效还会受到脂肪酸链长的影响^[4]^。鉴于不同油脂类药用辅料中脂肪酸种类和含量的特异性^[8]^,脂肪酸不仅可作为油脂类药用辅料质量评价的重要依据,还有望应用于不同类型油脂类药用辅料的快速鉴别和掺伪评价。因此,对多种脂肪酸进行灵敏、高效的定量检测,将有助于油脂类药用辅料的分析研究和质量提升。

目前多种分析技术已被应用于脂肪酸的分析,如薄层色谱法^[9]^、毛细管电泳法^[10]^、气相色谱法(GC)^[11⇓⇓-14]^、高效液相色谱法(HPLC)^[15,16]^、核磁共振波谱法^[17,18]^、红外光谱法^[19]^以及质谱法(MS)^[20⇓⇓-23]^等。2024版《英国药典》^[9]^采用薄层色谱法来鉴别脂肪油,方法操作简单,检测效率高,但其使用的特殊材料(十八烷基硅胶)在国内并不普及,且国产十八烷基硅胶的成本昂贵、结果重复性差,不利于实际应用。GC是最为常用的脂肪酸分析技术,其具有灵敏度高和分离效果好等优点,但由于部分脂肪酸在高温条件下不稳定且沸点较高,使用GC进行分析时通常需要先将油脂中的脂肪酸进行甲酯化^[24]^。然而,部分脂肪酸甲酯在前处理及分离分析过程中可能发生降解或异构化,从而影响GC的定量准确性^[25]^。LC技术也常被应用于脂肪酸的分离检测,但为提高检测灵敏度,一般也需要采用衍生化的方法。其他常见检测技术如红外光谱、核磁共振波谱等,它们的灵敏度相对较低且所需样品量较大,一般不适用于精准度需求较高的含量测定实验。MS技术虽具有高灵敏度和高选择性,但其方法开发及运行成本较高,且对仪器设备和人员操作水平的要求也较高。超临界流体色谱(SFC)使用处于临界温度及临界压力以上的流体(例如超临界CO_2_)作为流动相^[26]^,可在较低的温度下对沸点较高、热稳定性较差的脂肪酸类化合物进行分析^[27]^,能够避免复杂的衍生化反应,在简化前处理步骤的同时,也大幅缩短了样品处理及检测时间。作为一种通用型检测器^[28]^,蒸发光散射检测器(ELSD)能够实现脂肪酸的直接检测,极大地降低了反应过程中所引入的分析误差^[29]^;同时,ELSD能够与多种溶剂和梯度兼容,有助于分析速度的提升^[30]^。不同种类油脂所含的脂肪酸不尽相同,且部分脂肪酸之间存在结构相似、物理化学性质差异较小的问题,导致难以分离,而在SFC体系中,可以通过使用改性剂来改善不同脂肪酸之间的分离效果;并且,SFC不使用非挥发性溶剂(例如水)作为流动相,因此其能够与ELSD联用,从而提高检测灵敏度^[31]^。与GC相比,SFC-ELSD技术可以避免甲酯化等衍生化反应可能带来的不确定性,但目前尚未见使用SFC-ELSD对油脂类药用辅料中多种脂肪酸同时进行检测的相关报道。

油脂类药用辅料中的脂肪酸具有游离态和结合态两种存在形式,受环境影响两种形态可能会相互转化;同时由于游离态脂肪酸在油脂类药用辅料中的含量低,实际检测难度较大。2020版《中国药典》^[7]^对脂肪油中的脂肪酸组成检测过程中采用三氟化硼作为催化剂进行甲酯化处理,但甲酯化反应存在一系列问题,如反应过程繁琐耗时、甲酯化产物生成量不恒定、反应不完全及可能产生大量副产物等^[25]^。皂化反应是指油脂与碱性溶液之间的反应,它能够使油脂降解,从而释放出相应的脂肪酸。与甲酯化反应相比,皂化反应的处理过程更加简单、快捷,试剂成本也相对较低,更适合作为SFC-ELSD方法中脂肪酸检测的前处理手段。采用皂化反应将油脂类药用辅料中的结合态脂肪酸转化为游离态脂肪酸,可以有效解决游离态脂肪酸含量低及游离态与结合态相互转化所导致的油脂特征偏离问题。

基于上述问题,本研究将皂化反应作为前处理手段,利用SFC-ELSD技术,建立了同时测定肉豆蔻酸、棕榈酸、硬脂酸、花生酸、二十二烷酸和木蜡酸等6种脂肪酸的分析方法,并将该方法成功应用于5种不同油脂类药用辅料(玉米油、大豆油、椰子油、橄榄油、花生油)中6种脂肪酸的含量测定。同时,结合主成分分析,本方法能够实现对不同类型油脂类药用辅料的良好区分,为油脂类药用辅料的快速鉴别和质量控制提供了技术支撑,并有望应用于复杂体系中油脂类药用辅料的掺伪分析。

## 1 实验部分

### 1.1 仪器、试剂与材料

Agilent 1290 Infinity Ⅱ超临界流体色谱仪和蒸发光散射检测器(美国Agilent公司);甲醇、乙腈、正己烷、异丙醇(均为色谱纯)、Milli-Q超纯水机购自德国Merck公司;氢氧化钠、乙酸铵均为分析纯,购自国药集团化学试剂有限公司;所有实验用水均为超纯水。

6种脂肪酸标准物质:肉豆蔻酸(C_14_H_28_O_2_,批号190162-201501)、棕榈酸(C_16_H_32_O_2_,批号190029-201904)、硬脂酸(C_18_H_36_O_2_,批号190032-201603)对照品均购自中国食品药品检定研究院;花生酸(C_20_H_40_O_2_,批号506-30-9,纯度99%)、木蜡酸(C_24_H_48_O_2_,批号557-59-5,纯度>99%)标准品购自阿拉丁试剂(上海)有限公司;二十二烷酸(C_22_H_44_O_2_,批号112-85-6,纯度95%)标准品购自上海毕得医药科技股份有限公司。

5种油脂类药用辅料:玉米油(批号8001-30-7,化学纯)购自上海麦克林生化科技股份有限公司;大豆油(批号8001-22-7,化学纯)、椰子油(批号8001-31-8,化学纯)、橄榄油(批号8001-25-0,化学纯)、花生油(批号8002-03-7,化学纯)均购自阿拉丁试剂(上海)有限公司。

### 1.2 标准储备液和混合标准溶液的配制

准确称取各脂肪酸标准物质0.1 g(精确至0.0001 g)至10 mL容量瓶中,分别用正己烷溶解并配制成10 mg/mL的6种脂肪酸标准储备液,放入4 ℃冰箱中避光保存,备用;分别吸取各脂肪酸标准储备液100 μL于1 mL棕色容量瓶中,用异丙醇稀释至刻度,得到1 mg/mL的混合标准溶液,放入4 ℃冰箱中避光保存,备用。

### 1.3 样品溶液制备

准确称取样品0.1 g(精确至0.0001 g),置于50 mL回流瓶中,加入4 mL 0.5 mol/L的氢氧化钠甲醇溶液进行皂化反应,在100 ℃水浴中加热回流至油滴消失(通常约10 min);待溶液冷却至室温后,向回流瓶中加入4 mL超纯水,混合均匀后用7 mol/L盐酸水溶液将pH值调至2~4,之后将溶液转移至分液漏斗中,加入正己烷萃取两次,每次3 mL,合并正己烷层;再用超纯水萃取正己烷相两次,每次3 mL,合并正己烷层;取250 μL正己烷相溶液,用氮气吹干,再加入异丙醇复溶至1 mL,进样测定。

### 1.4 色谱条件

色谱柱:Viridis HSS C_18_ SB柱(100 mm×3 mm, 1.8 μm,美国Waters公司);流动相A为超临界CO_2_,流动相B为20 mmol/L乙酸铵甲醇溶液;柱温35 ℃;流速1.2 mL/min;背压设定为9 MPa;进样室温度10 ℃;进样量3 μL。梯度洗脱程序:0~6 min, 3%B~20%B; 6~9 min, 20%B~10%B; 9~10 min, 10%B~3%B; 10~12 min, 3%B。补偿泵溶剂为甲醇,流速0.3 mL/min。ELSD检测器设定条件:蒸发管温度30 ℃,雾化温度60 ℃,载气为高纯氮气,载气流速1.4 L/min。

## 2 结果与讨论

### 2.1 脂肪酸分析条件的优化

#### 2.1.1 色谱条件的优化

各个脂肪酸之间的结构具有相似性,因此通过优化色谱分离条件来改善脂肪酸的分离度是非常必要的。实验考察了3种色谱柱(Viridis HSS C_18_ SB色谱柱(100 mm×3 mm, 1.8 μm)、Viridis BEH 2-Ethylpyridine色谱柱(100 mm×2.1 mm, 1.7 μm)、Viridis BEH色谱柱(100 mm×3 mm, 1.7 μm))对6种脂肪酸分离效果的影响。如图1a所示,仅当采用HSS C_18_ SB色谱柱进行分离时,6种脂肪酸均能实现基线分离且色谱峰峰形良好,无明显延展或拖尾现象。这可能归因于脂肪酸的羧基与HSS C_18_ SB色谱柱固定相上极性活性位点(硅烷醇基团)之间的强相互作用。因此,本实验选择Viridis HSS C_18_ SB色谱柱用于6种脂肪酸的分离。

**图 1:**
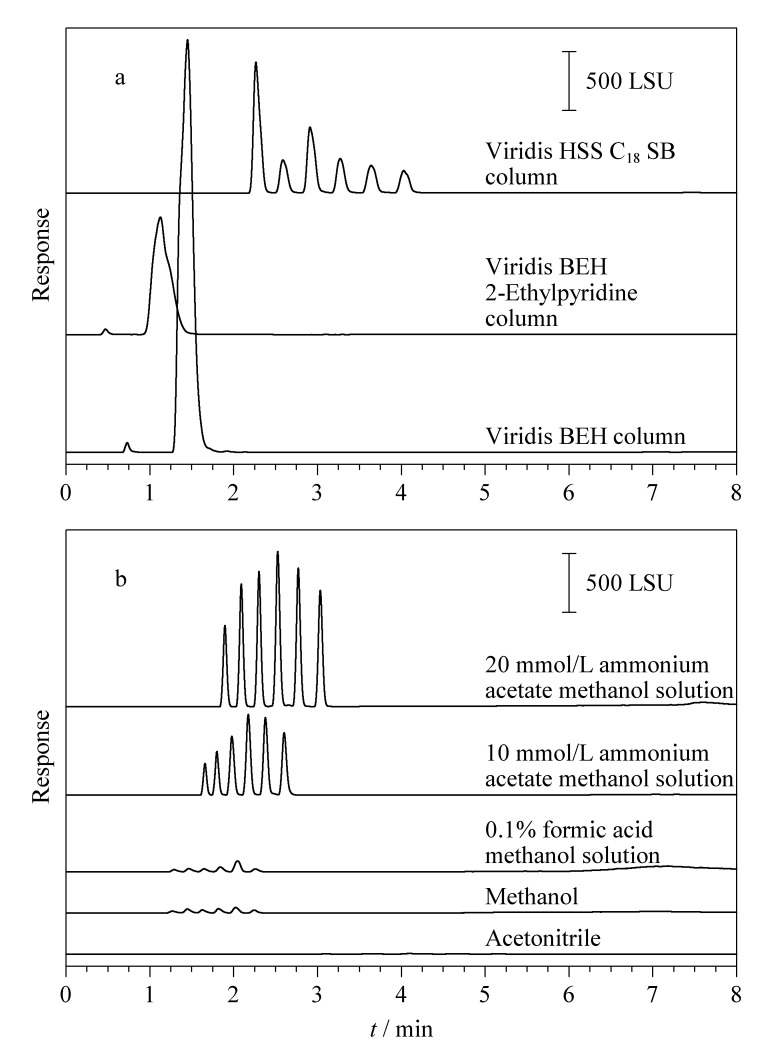
不同(a)色谱柱及(b)流动相条件下6种脂肪酸的色谱图

CO_2_具有化学惰性、经济、易得等优点,是SFC最常用的流动相,但其极性较小,在分析极性较大的化合物时,常需与有机溶剂搭配使用;同时也可以在有机溶剂中加入适当比例的改性剂(如乙酸铵),以改善峰形、提高分离度、增加检测灵敏度^[32]^。实验分别考察了乙腈、甲醇、0.1%甲酸甲醇溶液、10 mmol/L乙酸铵甲醇溶液和20 mmol/L乙酸铵甲醇溶液对6种脂肪酸分离效果和响应强度的影响。如图1b所示,与乙腈相比,甲醇更有利于脂肪酸的洗脱和检测;与0.1%甲酸甲醇溶液和10 mmol/L乙酸铵甲醇溶液相比,在20 mmol/L乙酸铵甲醇溶液流动相条件下,6种脂肪酸的分离度和响应强度最好。因此,实验最终采用超临界CO_2_和20 mmol/L乙酸铵甲醇溶液作为流动相。

柱温、背压和流速是影响目标分析物保留行为的重要因素。实验考察了不同柱温(35、45、55 ℃)、背压(9、10、11 MPa)和流速(0.8、1.0、1.2 mL/min)对6种脂肪酸之间分离度的影响,结果如图2所示。随着柱温及背压的升高,6种脂肪酸之间的分离度降低(图2a和图2b);而随着流速的增加,大部分脂肪酸之间的分离度逐渐增加。综合考虑多个因素及仪器参数设置范围,最终将柱温、背压和流速分别设定为35 ℃、9 MPa和1.2 mL/min。

**图 2:**
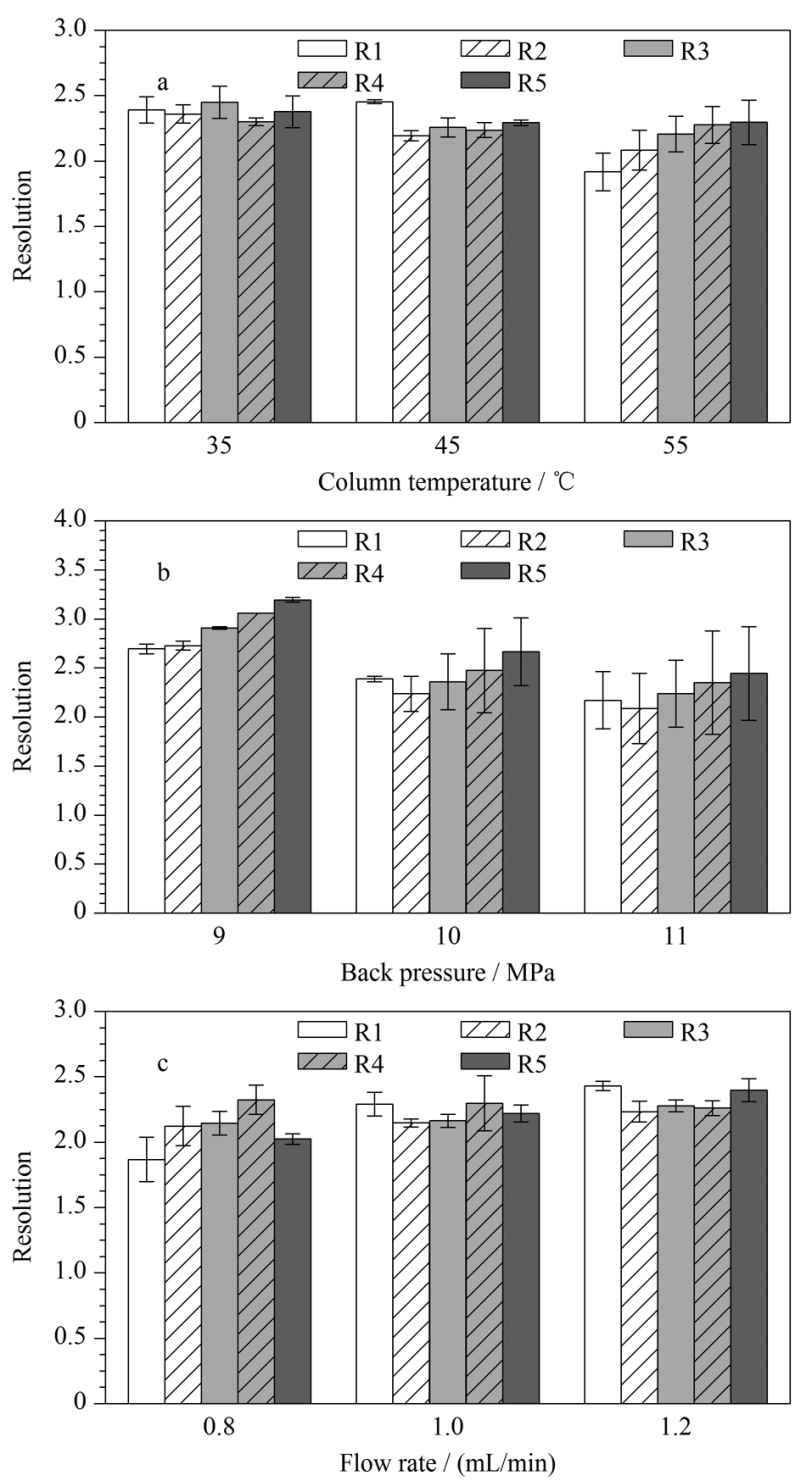
不同(a)柱温、(b)背压和(c)流速对6种脂肪酸 之间分离度的影响(*n*=3)

#### 2.1.2 ELSD参数的优化

ELSD中的两个关键参数分别是蒸发管温度和雾化器载气流速^[33]^。雾化器载气流速直接决定了所形成的分析物粒子半径大小和光散射能力,进而影响目标分析物的响应强度;蒸发管温度会对检测器内的流动相蒸发情况产生影响,过高或过低的温度都会影响目标物的信噪比。实验分别考察了不同蒸发管温度(30、40、50、60、70 ℃)及雾化器载气流速(1.1、1.2、1.4、1.6、1.8 L/min)对脂肪酸响应强度的影响。如图3a所示,随着蒸发管温度上升,6种脂肪酸的响应强度及其总和均呈明显的下降趋势;如图3b所示,随着载气流速增加至1.8 L/min, 6种脂肪酸的响应强度略有下降,但在实际分析过程中,当雾化器载气流速低于1.4 L/min时,脂肪酸定量检测的重复性较差,这可能是载气流速过低时,所进入蒸发管的液滴较大、蒸发效率不稳定导致的。综上,ELSD参数确定为蒸发管温度30 ℃,雾化器载气流速1.4 L/min。采用优化后的分析条件对6种脂肪酸混合标准溶液进行测定,色谱图如图4所示,6种脂肪酸实现了良好分离。

**图 3:**
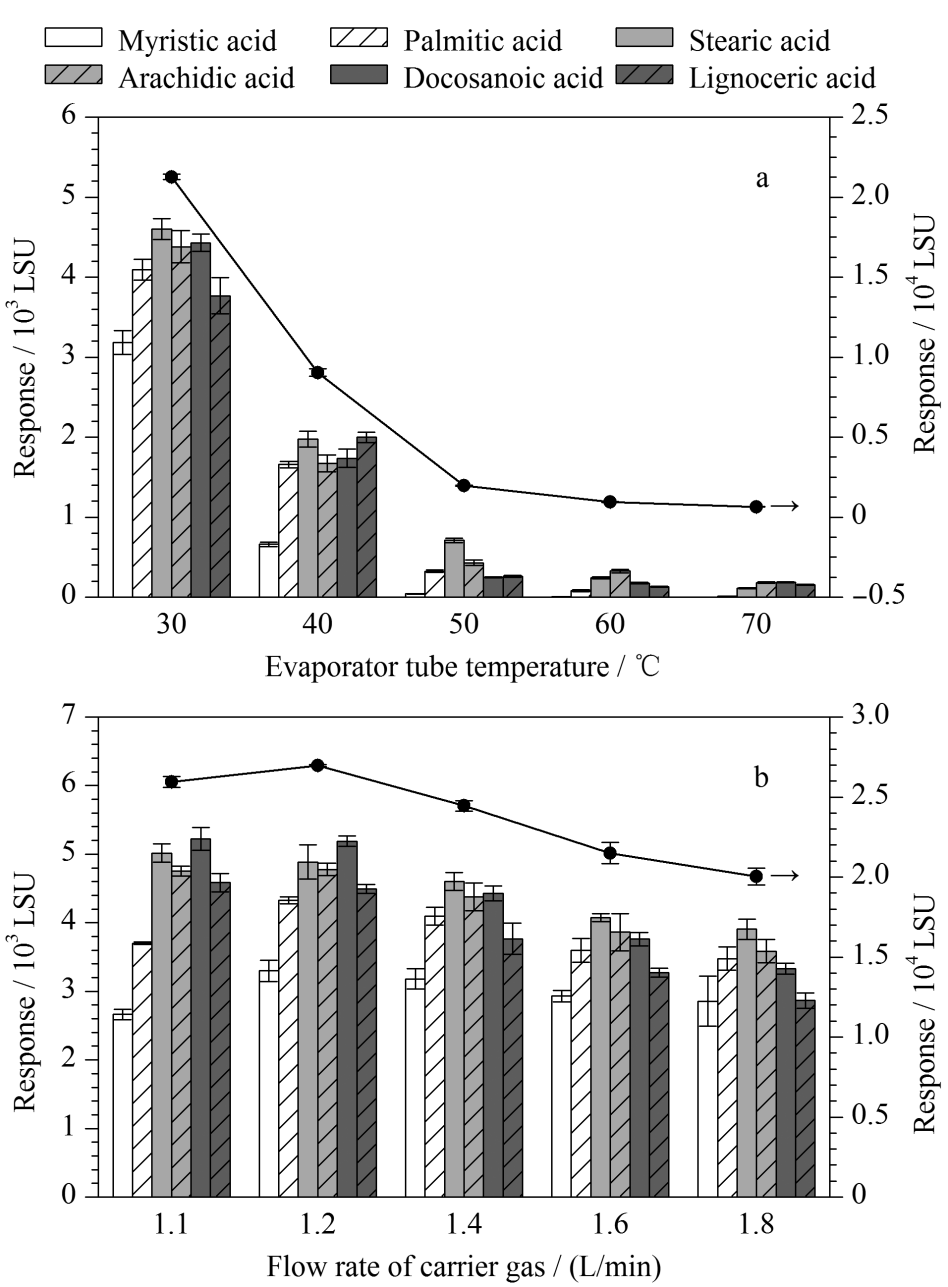
不同(a)蒸发管温度及(b)雾化器载气流速对6种 脂肪酸响应强度的影响(*n*=3)

**图 4:**
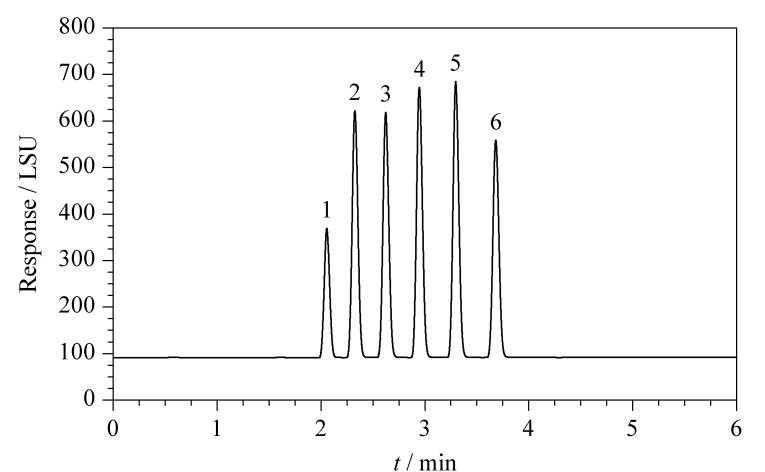
优化条件下6种脂肪酸混合标准溶液(100 mg/L)的色谱图

### 2.2 方法学验证

#### 2.2.1 专属性与系统适用性

采用所建立方法分别对5种油脂类药用辅料(玉米油、大豆油、椰子油、橄榄油和花生油)样品进行检测,结果见附图1(详见www.chrom-China.com)。实验结果显示,5种样品溶液中各目标峰之间的分离度良好,说明本方法的专属性良好。按照2020版《中国药典》^[7]^(通则0512)分别对混合标准溶液中6种脂肪酸色谱峰的理论塔板数、分离度和拖尾因子进行计算,结果见附表1, 6种脂肪酸色谱峰的理论塔板数为7322~16279,分离度均大于3.75,拖尾因子为1.05~1.10,均满足实验要求。

#### 2.2.2 进样精密度与脂肪酸稳定性

将脂肪酸混合标准溶液连续进样6次,计算相对标准偏差(RSD),结果表明,6种脂肪酸的RSD均不大于3.65%,进样精密度良好。实验以花生油为例来考察6种脂肪酸的稳定性:精密称取花生油样品,向其中加入6种脂肪酸混合标准溶液,之后分别在第0、2、4、8、10、24、48 h进行测定,记录各个脂肪酸的峰面积,并计算各时间点之间6种脂肪酸峰面积的RSD;结果表明,6种脂肪酸的RSD均不大于5.49%,说明实际样品中6种脂肪酸在48 h内均能保持稳定。

#### 2.2.3 线性范围、检出限及定量限

用异丙醇将6种脂肪酸混合标准溶液稀释成质量浓度分别为5、10、25、50、100、200、250、500 mg/L的系列混合标准工作溶液,如表1所示,以各个脂肪酸的峰面积为纵坐标(*y*)、质量浓度为横坐标(*x*)绘制标准曲线。结果表明,6种脂肪酸在各自的线性范围内线性关系良好。取各脂肪酸线性范围最低值及以下的系列混合标准工作溶液进行逐级稀释,进样并记录色谱图,分别以信噪比(*S/N*)为3和10时所对应的质量浓度作为检出限和定量限,结果表明,6种脂肪酸的检出限和定量限分别为5~10 mg/L和10~25 mg/L,满足检测要求。

**表 1:** 6种脂肪酸的线性回归方程、决定系数、线性范围、检出限和定量限

Compound	Linear regression equation	R^2^	Linear range/(mg/L)	LOD/(mg/L)	LOQ/(mg/L)
Myristic acid	y=10.308x-466.94	0.9910	25-500	10	25
Palmitic acid	y=14.791x-223.71	0.9951	25-500	10	25
Stearic acid	y=17.819x-101.65	0.9927	10-500	5	10
Arachidic acid	y=21.075x-205.81	0.9955	10-250	5	10
Docosanoic acid	y=19.853x-207.61	0.9924	10-250	5	10
Lignoceric acid	y=17.987x-211.35	0.9909	10-250	5	10

*y*: peak area; *x*: mass concentration, mg/L.

#### 2.2.4 加标回收率及精密度

为排除基质中的脂肪酸干扰,利用本文所建立方法对花生油样品中6种脂肪酸的含量进行测定,结果如表2所示。之后以确定了脂肪酸含量的花生油样品为基质,分别添加低、中、高3个水平(50、100、250 mg/L)的6种脂肪酸混合标准溶液,每个加标水平平行测定3次,计算加标回收率和RSD。结果如表2所示,6种脂肪酸在3个加标水平下的回收率为80.93%~111.66%, RSD为0.49%~8.29%,方法准确度和精密度均能够满足测定需求,可用于实际样品分析。

**表 2:** 6种脂肪酸在3个加标水平下的回收率和精密度(*n*=3)

Compound	Background/ (mg/L)	Spiked level/ (mg/L)	Measured/ (mg/L)	Recovery/ %	RSD/ %
Myristic	-	50	42.07	84.13	3.98
acid		100	83.51	83.51	2.58
		250	224.81	89.92	3.84
Palmitic	147.54	50	200.93	106.78	4.58
acid		100	257.89	110.35	2.62
		250	386.20	95.46	1.19
Stearic	42.35	50	88.73	92.76	3.35
acid		100	136.18	93.83	6.64
		250	244.68	80.93	0.49
Arachidic	27.16	50	81.36	108.40	2.86
acid		100	133.51	106.35	5.00
		250	234.05	82.76	3.05
Docosanoic	46.33	50	102.16	111.66	3.51
acid		100	151.36	105.03	2.44
		250	277.56	92.49	2.37
Lignoceric	25.73	50	77.56	103.65	6.11
acid		100	126.99	101.26	3.60
		250	278.84	101.24	8.29

-: not detected.

### 2.3 与2020版《中国药典》^[7]^(通则0713)方法比较

2020版《中国药典》^[7]^(通则0713)中采用GC方法来检测油脂中的脂肪酸组成。采用2020版《中国药典》^[7]^(通则0713)方法和本文所建立方法分别对花生油样品中的6种脂肪酸含量进行检测,两种方法均未检测到肉豆蔻酸,将检测结果进行比较,结果见图5。在两种方法中,大部分脂肪酸含量的检测结果相近,但棕榈酸和硬脂酸含量的检测结果存在一定程度的偏差,这可能与两种方法所采用的检测器(ELSD和氢火焰离子化检测器(FID))原理和灵敏度不同有关。此外,由于油脂中的脂肪酸沸点较高,采用GC方法需要对油脂样品进行甲酯化处理,而本方法则可直接用于脂肪酸的检测;检测器和目标分析物的不同以及甲酯化反应的不确定性都可能会对检测结果产生影响^[34]^。在6种脂肪酸中,棕榈酸含量变化幅度受反应条件的影响最大^[35]^,然而在SFC-ELSD方法中,这种现象不会对各种油脂类药用辅料的鉴别造成干扰。

**图 5:**
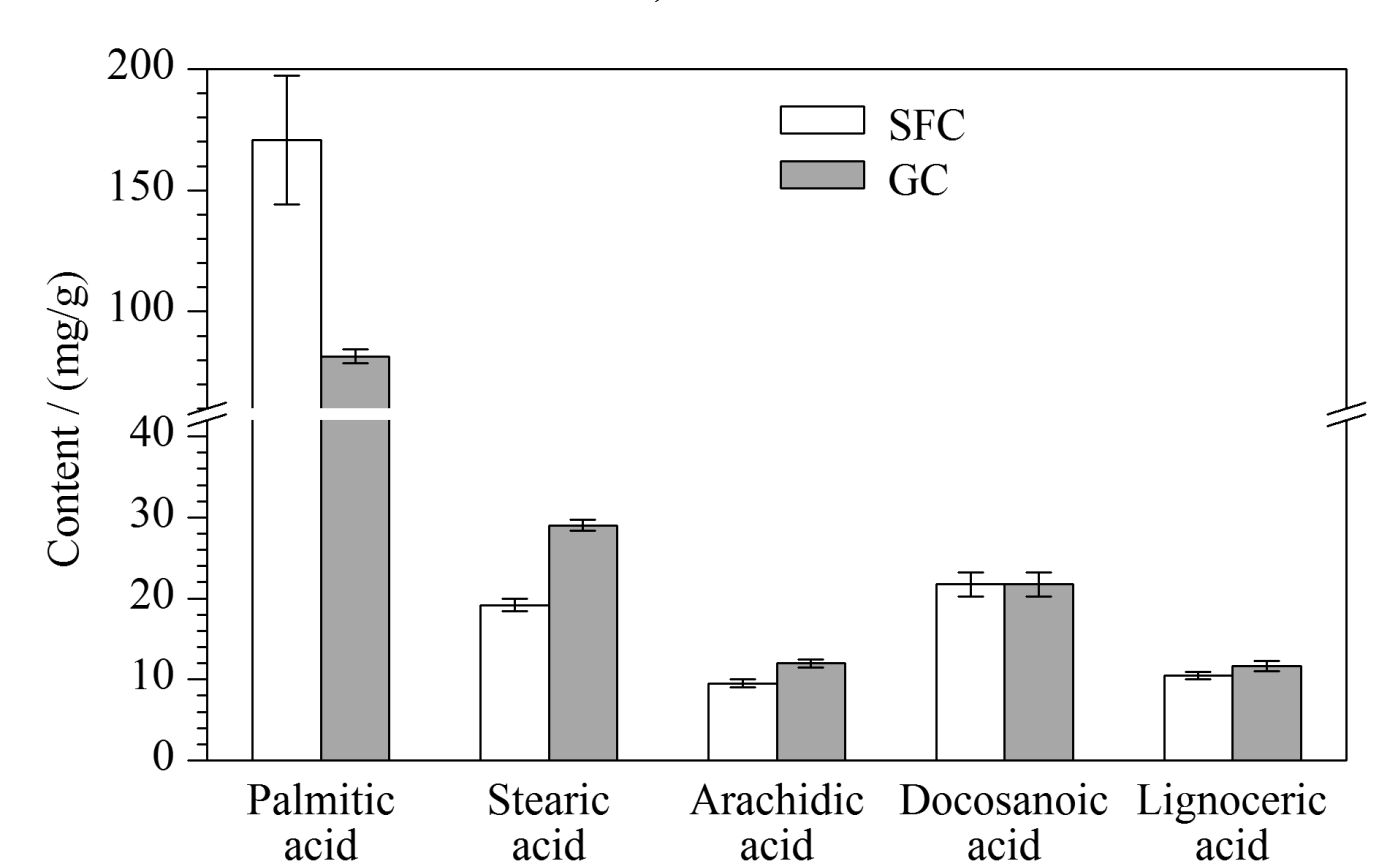
两种方法对5种脂肪酸含量检测结果的比较(*n*=3)

### 2.4 实际样品的检测及掺伪鉴别

采用所建立方法对5种常见油脂类药用辅料(玉米油、大豆油、椰子油、橄榄油和花生油)中的6种脂肪酸进行检测,每种样品平行制备3份,结果如图6所示。椰子油中仅检测到肉豆蔻酸、棕榈酸、硬脂酸3种脂肪酸;玉米油、大豆油、橄榄油中均检出棕榈酸、硬脂酸、花生酸和二十二烷酸4种脂肪酸,且棕榈酸的含量最高;花生油中共检出5种脂肪酸,分别为硬脂酸、棕榈酸、花生酸、二十二烷酸和木蜡酸。在玉米油、大豆油和橄榄油中,花生酸和二十二烷酸的含量较低,而在花生油中二十二烷酸的含量高于其他3种脂肪酸(硬脂酸、花生酸和木蜡酸)。5种油脂类药用辅料中,仅椰子油检出肉豆蔻酸,且肉豆蔻酸、棕榈酸和硬脂酸的含量随碳原子数增加逐渐降低,这与2020版《中国药典》^[7]^记载情况基本一致。实验结果说明,该方法可用于分析不同油脂类药用辅料中的脂肪酸组成,而所得到的脂肪酸组成特征又为油脂类药用辅料的区分提供了可能。

**图 6:**
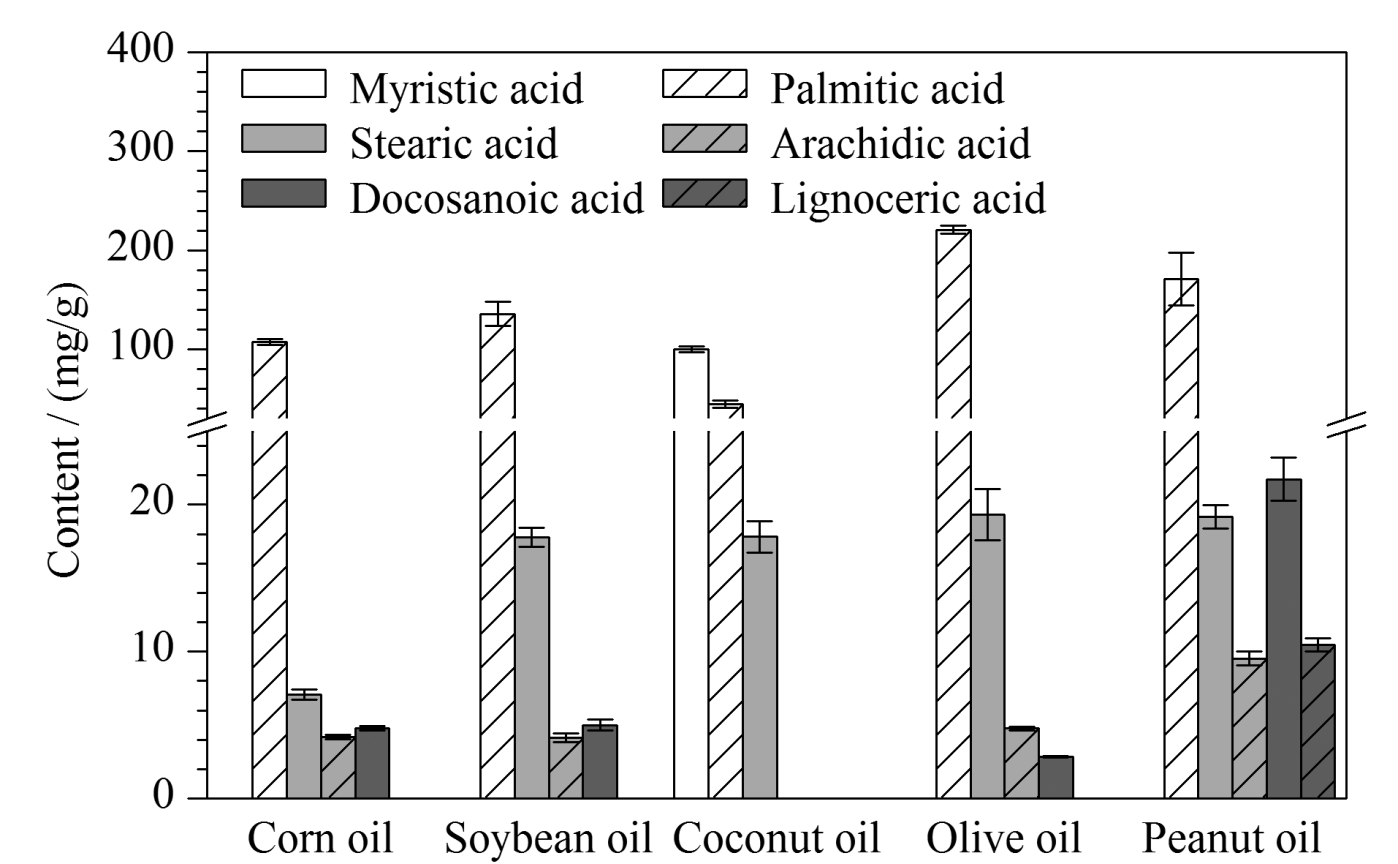
5种油脂类药用辅料中6种脂肪酸的含量测定(*n*=3)

以5种油脂类药用辅料中各脂肪酸的含量为特征变量,根据累积贡献率将得分进行排序,选取前两个主成分绘制5种油脂类药用辅料的主成分分析(PCA)散点图。结果如图7a所示,第一个主成分(PC1)和第二个主成分(PC2)的累计贡献率之和为78.56%。一般认为,当累计贡献率大于60%时,PCA模型可以作为分离模型^[36]^。从图7a中可以看出,5种油脂类药用辅料的区分度良好,说明本研究所选择的6种脂肪酸可作为5种油脂类药用辅料的区分指标。花生油的聚类簇与其他油脂之间的距离较远,说明其脂肪酸组成及含量与其他油脂存在较大差异;椰子油的聚类簇单独聚于右下角,说明其脂肪酸组成与其他4种油脂类药用辅料具有明显不同。橄榄油具有丰富的营养成分和保健价值,市面价格较高,因此使用低价植物油进行掺假的现象频繁发生。分别向橄榄油中加入不同体积分数的花生油,建立橄榄油掺伪模型(橄榄油体积分数分别为90%、80%、60%、50%),并利用PCA散点图进行掺伪判别,结果如图7b所示。

**图 7:**
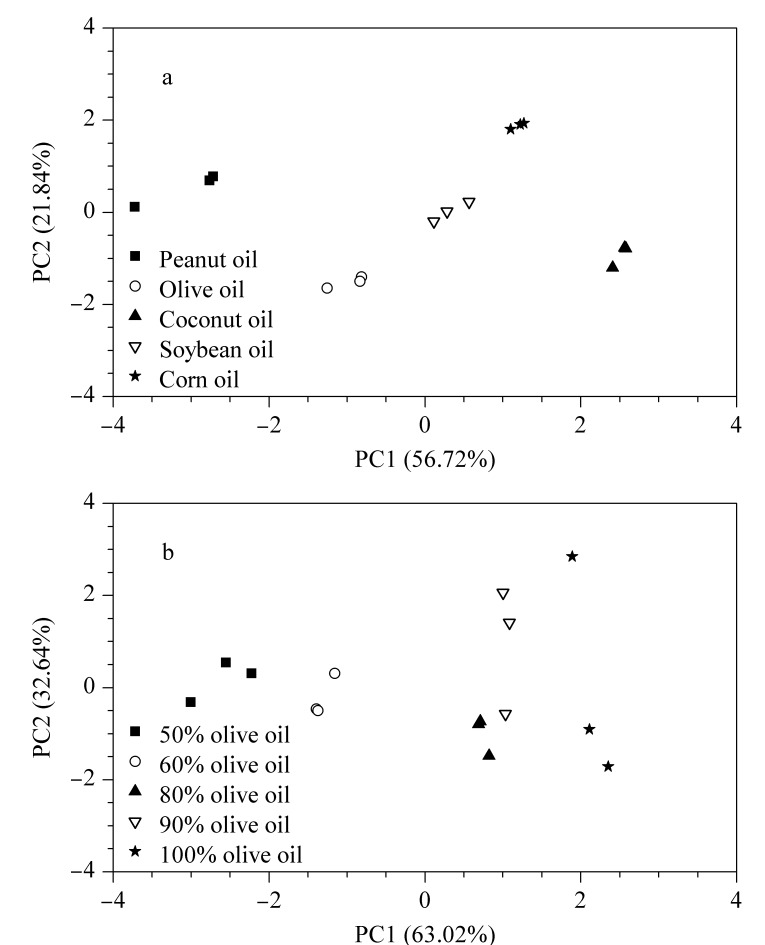
(a)5种油脂类药用辅料和(b)橄榄油掺伪样品的PCA得分图

随着橄榄油体积分数的不断下降,掺伪样品与纯正橄榄油样品之间的区分度逐渐明显;当掺伪体积分数为10%及以上时,掺伪样品与纯正橄榄油均可通过PCA模型进行很好的区分。油脂类药用辅料中脂肪酸的组成复杂,含量范围波动较大,因此对所有脂肪酸进行精确表征的难度较大。测定油脂类药用辅料中脂肪酸组成及含量的目的主要是用于确定和区分油脂种类,将6种脂肪酸作为目标分析物,有望实现油脂类药用辅料的准确鉴别。在不同方法比较中,各脂肪酸的测定含量虽然存在偏差,但在实际应用中发现,测定结果不会对油脂种类鉴别产生影响。

## 3 结论

本方法采用皂化反应作为前处理手段,利用SFC-ELSD技术,建立了同时测定肉豆蔻酸、棕榈酸、硬脂酸、花生酸、二十二烷酸和木蜡酸等6种脂肪酸的分析方法。该方法能够减少常规衍生化过程的繁琐步骤及高成本试剂的使用,同时也避免了衍生化过程中造成的样品损失和检测误差,提高了油脂类药用辅料中脂肪酸的检测灵敏度。通过对分析条件及检测器参数进行优化,该方法能够在12 min内实现6种脂肪酸的分离和定量分析,并成功应用于5种常见油脂类药用辅料(玉米油、大豆油、椰子油、橄榄油和花生油)中6种脂肪酸的检测。该方法灵敏度高,重复性和稳定性好,能够满足油脂类药用辅料中脂肪酸的分析要求,为灵敏检测油脂类药用辅料中的脂肪酸提供了一种新的技术手段,同时也为油脂的合理选择、质量控制和掺伪鉴别提供了参考。
